# New data analysis for BioSAXS at the ESRF

**DOI:** 10.1107/S1600577522007238

**Published:** 2022-08-23

**Authors:** Jérôme Kieffer, Martha Brennich, Jean-Baptiste Florial, Marcus Oscarsson, Alejandro De Maria Antolinos, Mark Tully, Petra Pernot

**Affiliations:** a ESRF – The European Synchrotron, 71 Avenue des Martyrs, 38000 Grenoble, France; b European Molecular Biology Laboratory, 71 Avenue des Martyrs, 38000 Grenoble, France; NSRRC, Taiwan

**Keywords:** BioSAXS, online data analysis, solution scattering, proteins, biological small-angle X-ray scattering, automation, high brilliance, structural biology, high-throughput SAXS, size-exclusion chromatography, online purification

## Abstract

The second phase of the ESRF upgrade program did not only provide a new storage ring (Extremely Brilliant Source, EBS) but also allowed several beamlines to be refurbished. The BioSAXS beamline (located on port BM29) was upgraded with a new wiggler source and a larger detector. All analysis software has been rewritten to cope with the increased data flux and continues to provide beamline users with reduced and pre-processed data in real time. This article describes *FreeSAS*, an open-source collection of various small-angle scattering analysis algorithms needed to reduce and analyze BioSAXS data, and *Dahu*, the tool used to interface data analysis with beamline control. It further presents the data-processing pipelines for the different data acquisitions modes of the beamline, using either a sample changer for individual homogeneous samples or an inline size-exclusion chromatography setup.

## Abstract 2

A detailed presentation of the automatic data analysis pipelines for the BioSAXS beamline at the European synchrotron.

## 1. Introduction

Small-angle scattering (SAS) provides information on the shape of macromolecules on the nanometre scale and is particularly suited for biological samples thanks to a large range of suitable buffer conditions. Unlike single-crystal diffraction or nuclear magnetic resonance (NMR) which offer atomic resolution, BioSAS provides only information on the envelope of macro­molecules: it can be used to validate the relative position of large structures in the assembly of biological complexes (Schroer & Svergun, 2018 ▸). Structural biologists perform small-angle scattering (SAXS) experiments to validate the size and the shape of their protein or complex under study. Since most beamline users are biologists, they are neither synchrotron nor SAXS specialists. A full analysis of their data is thus of crucial importance to them (Basu *et al.*, 2019 ▸).

Whereas most BioSAXS beamlines throughout the world have focused on the graphical user interface for helping their users with data analysis (Basham *et al.*, 2015 ▸; Classen *et al.*, 2010 ▸), the BM29 beamline from the ESRF (Pernot *et al.*, 2013 ▸) has focused on fully automated data analysis which provides users not only with the reduced curves and pre-analyzed data but also with all metadata and parameters needed to reprocess their data and get them published according to the relevant guidelines (Trewhella *et al.*, 2017 ▸). BM29 had an automated pipeline for data analysis which was based on *EDNA* (Incardona *et al.*, 2009 ▸) and used the *ATSAS2* (Petoukhov *et al.*, 2012 ▸) software underneath. While the outcome of these processings was very appreciated by beamline users, the system was already close to the maximal throughput possible in terms of performances. The new EBS source (Extremely Brilliant Source; Chaize *et al.*, 2018 ▸) of the ESRF not only provides a higher brilliance but also new wiggler sources for the former bending-magnet beamlines which triggered the re-build and the upgrade of most of them. The BM29 BioSAXS beamline was rebuilt in 2019 and now features a two-pole wiggler source and a new Pilatus3 2M detector designed to be mounted in vacuum (previously 1M). This has lead to a substantial increase in data rate, mainly due to the larger detector.

This paper is divided into two main parts and starts with a presentation of the tools developed for online data analysis of SAXS data – *FreeSAS* and *Dahu*, the job scheduler. Section 3 presents the three different pipelines used at the beamline: the common pipeline for the reduction of scattering images, the pipeline used with the sample changer, and the one used with the size-exclusion chromatography setup.

## 2. Tools

During the upgrade of BM29 beamline, most software have been replaced by completely new developments: the sequencer *SPEC* (Swislow, 1987–2022 ▸) was replaced by *BLISS* (Guijarro *et al.*, 2020 ▸), the graphical user interface *BsxCuBE* (*BioSAXS Customized Beamline Environment*) was re-implemented with web-technology, replacing the *PyQt4* interface. To meet the real-time feedback requirement, the software performing the analysis was also re-written.

The precise benchmark of the execution times (see also Section 4.2) of the previous *EDNA*-based pipeline (Brennich *et al.*, 2016 ▸) demonstrated that time was mostly spent in launching external tools coming from the *ATSAS2* suite and in parsing output files produced by those tools, not in the execution of those external programs. It was decided to rewrite all pipelines in plain Python (van Rossum, 1989 ▸) and to regroup all SAS-related analysis within a library, *FreeSAS*, which would be easy to distribute. Pipelines (Kieffer, 2020–2022 ▸) are heavily optimized for running on the beamline hardware and thus cannot easily be reused despite their MIT licence. Finally, this code is interfaced to the control software, *BLISS* (Guijarro *et al.*, 2020 ▸), via *TANGO* (Götz *et al.*, 2003 ▸) and uses a simple task scheduler, *Dahu*, already used at the TRUSAXS beamline (Narayanan *et al.*, 2022 ▸).

### 2.1. Small-angle scattering analysis tools, *FreeSAS*

*FreeSAS* is a Python library (van Rossum, 1989 ▸) containing SAS analysis tools available both via a Python API and from the command line interface. It does not claim to be as complete as the *ATSAS* counterpart (Manalastas-Cantos *et al.*, 2021 ▸), but is free, released under the liberal MIT licence (*i.e.* it can be included into commercial products), all source code is available publicly on github (Kieffer *et al.*, 2021 ▸), and it is open to external contributions. Table 1 ▸ summarizes this comparison. Despite Python being an interpreted language, *FreeSAS* is performance-oriented and most of the processing is performed via Cython (Behnel *et al.*, 2011 ▸) extensions, written in C and compiled, to obtain the required performances. *FreeSAS* has been made available and packaged independently from *Dahu* (the online analysis tools) and from the processing pipelines so that scientists can reprocess their data and compare their results with those of other analysis software like *ScÅtter* (Rambo, 2017 ▸). The current release, *FreeSAS 0.9*, supports Python 3.6 to 3.9.

#### 2.1.1. SAS plotting

**Figure 1:**
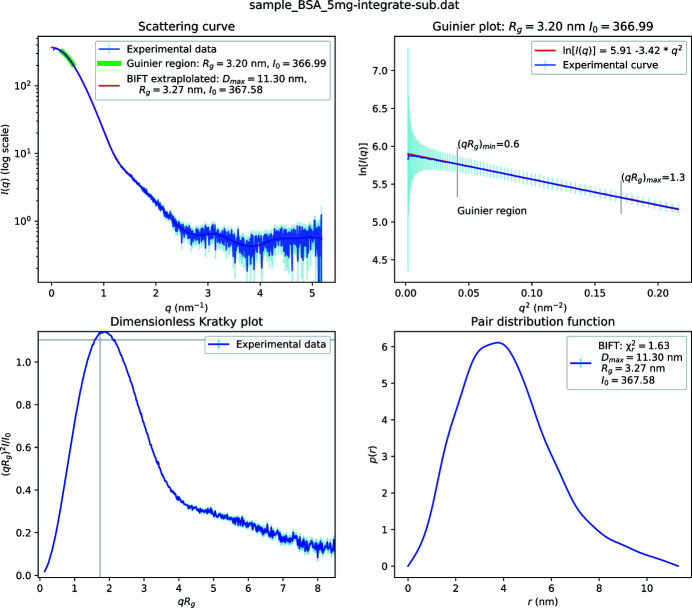
Default visualization of BioSAS data using the *FreeSAS* tool on a three-column (*q*, *I* and σ) ASCII file – here bovine serum albumin (BSA) acquired with 12.5 keV photons. The top-left plot presents the classical *I* = *f*(*q*) plot on a semi-logarithmic scale with the Guinier region highlighted in green and IFT-based curve superimposed in red. Those results come from the Guinier analysis (top right) based on a linear fit of log(*I*) = *f*(*q* ^2^) and from the pair distribution function [*p* = *f*(*r*)] obtained from the BIFT analysis (bottom right). A dimensionless Kratky plot is presented in the lower-left plot.

The *FreeSAS* command line tool (provided by the *FreeSAS* suite), Fig. 1 ▸, provides a way to plot a semi-logarithmic representation of the SAS curve: *I* = *f*(*q*), where *q* = 4πsin(θ)/λ is the amplitude of the scattering vector, θ is half the scattering angles, λ the wavelength and *I* the recorded intensity at a given *q*, alongside some basic analyses which are described in the next sections. The program takes as entry a three-column ASCII file containing *q*, *I* and σ.

#### 2.1.2. Guinier-region fitting

The first analysis performed on BioSAS data is to determine the radius of gyration, *R*_g_, of the solvated macro­molecule and the forward-scattering intensity, *I*_0_ (Guinier & Fournet, 1955 ▸). Based on the Taylor expansion of the scattering curve at *q* = 0, *R*_g_ is obtained from the the linear regression of log[*I*(*q*)] = log(*I*_0_) − 1/3(*qR*_g_)^2^ on the proper *q*-range, at small angles, called the Guinier region. The selection of the Guinier region is far from obvious due to the beam-stop shadow and aggregation effects, and its upper limit depends on *R*_g_ itself: *q* *R*_g_ < 1.3. Thus multiple implementations are provided: 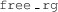 , which derives from the implementation in *BioXTAS-RAW* (Nielsen *et al.*, 2009 ▸); 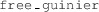 , which searches for a consensus region rather than the best possible Guinier region; and finally 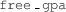 , which performs a Guinier-peak analysis (Putnam, 2016 ▸). The latter is a quick assessment of *R*_g_ and *I*_0_, sometimes less robust than the two other implementations, but suitable when many data sets are to be analyzed like in chromatography mode. It is worth mentioning that none of the three algorithms provide the exact same results as the original *AUTORG* (Petoukhov *et al.*, 2012 ▸) version from *ATSAS*. This highlights the importance of publishing the actual implementation of the algorithms with the associated numerical parameters used in it.

#### 2.1.3. Pair distribution function

Although the scattering curve *I*(*q*) is the Fourier transform of the pair distribution function *p*(*r*), the latter cannot directly be obtained from an inverse Fourier transform (IFT) due to the loss of phase information and the limited amount of information in the scattering curve. This ill-posed mathematical problem has no exact solution and is usually inverted using some extra constraints, like the finite size of the macro­molecule (defined by its maximum diameter, *Dmax*). *FreeSAS* proposes an IFT based on Bayesian statistics and derived from BIFT (Vestergaard & Hansen, 2006 ▸) and implementation in *BioXTAS-RAW* (Hopkins *et al.*, 2017 ▸). The implementation in *FreeSAS*, called 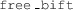 , is based on Cython (Behnel *et al.*, 2011 ▸) and uses BLAS (Blackford *et al.*, 2002 ▸) and multi-threading for performances. Despite their different approaches, 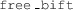 provides similar results to those of *DATGNOM* (Petoukhov *et al.*, 2007 ▸) from *ATSAS* which uses a Tikhonov regularization.

#### 2.1.4. Other tools available in *FreeSAS*

(i) 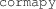 evaluates radiation damage by comparing couples of frames using the correlation-map (*CorMap*) algorithm implemented from the publication by Franke *et al.* (2015 ▸).

(ii) 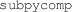 rotates and flips bead models to overlay them prior to merging them (Brennich *et al.*, 2016 ▸).

(iii) 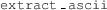 generates three-column [*q*, *I*_avg_ and σ(*I*)] text files (with headers) for compatibility with third-party tools. Following ESRF’s data policy (Dimper *et al.*, 2019 ▸), the default file format used at the BioSAXS beamline has changed to the hierarchical data format, HDF5 (The HDF Group, 2000–2021 ▸), for acquisition, analysis and archival of data. This is part of the FAIR principle (Wilkinson *et al.*, 2016 ▸) where the data portal (ESRF, 2011–2022 ▸) provides **F**indability, HDF5 provides **A**ccessibility, 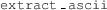 provides **I**nteroperability, hoping this data is **R**eused after the embargo period.

Table 1 ▸ provides some equivalence for program names provided by *FreeSAS* and *ATSAS* packages. The latter toolbox is built on top of several decades of experience acquired by the EMBL-Hamburg group and features some 60 programs to perform BioSAS data analysis. *FreeSAS* cannot compete on the number of features but tries to offer a clean Python interface to build pipelines in an efficient manner and fully open (both for usage and modification).

### 2.2. The job manager: *Dahu*

The role of the job manager is to ensure all processing requested by the client (here *BsxCuBE3*, the graphical user interface) are actually performed, informs the client about the finished jobs and warns it in case of an error.

*EDNA* was used as workflow manager for the previous data-analysis pipeline on several protein crystallography beamlines (Incardona *et al.*, 2009 ▸) and for the BioSAXS beamline at the ESRF (Brennich *et al.*, 2016 ▸). The parallelization model used in *EDNA* is based on Python threads and forking processes which was wasting resources in serializing and inter-process communication.

The *Dahu* job manager was designed for the low latency constraints of the TRUSAXS beamline (Narayanan *et al.*, 2022 ▸), where some jobs need to be processed within a dozen of milliseconds. Batch queuing systems, like *SLURM* (Yoo *et al.*, 2003 ▸), are very efficient at distributing heavy jobs, but none was optimized for reduced scheduling time (or low latency).

The *Tango* interface (Götz *et al.*, 2003 ▸) implemented in *Dahu* was kept similar to the one in *EDNA* in order to ease the transition. The scheduling of jobs is performed via a shared queue and only a few workers are running simultaneously in different threads. Thus the code runs actually in parallel only in sections where the Global Interpreter Lock from Python (GIL) is released, like in Cython extensions (Behnel *et al.*, 2011 ▸) from *FreeSAS* or in the OpenCL code from *pyFAI* (Kieffer & Ashiotis, 2014 ▸).

#### 2.2.1. *Dahu* job

The *Dahu* job manages the execution of one Dahu plugin (see next subsection) and provides a unique identifier which gives access to the status and output of the processing. Jobs see their input and output saved onto disk, which allows offline reprocessing in case of an issue during online data analysis.

#### 2.2.2. *Dahu* plugins

*Dahu* plugins implement the processing logic of the different pipelines. Written in simple Python and fairly independent of the *Dahu* framework, those plugins are often written or modified by beamline scientists themselves.

#### 2.2.3. Offline re-processing

Offline re-processing is made possible by the 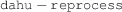 command-line tool. This tool was designed to (re-)execute one or several jobs based on the JSON description file (Pezoa *et al.*, 2016 ▸) saved by the online data analysis server. Since *Dahu* has virtually no dependencies, it can be deployed on any computer to reprocess data. Nevertheless, to reprocess data acquired at the BioSAXS beamline, one would need *FreeSAS* and all the other dependencies of the BioSAXS plugins, which are documented in the 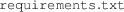 file in the plugin directory available at GitHub (Kieffer, 2020–2022 ▸).

## 3. Data analysis pipelines

There are two main experiments performed at the BioSAXS beamline – using the sample changer (referred to as SC) or the inline-chromatography setup (referred to as HPLC since it uses a high-pressure liquid chromatograph). Thus, two analysis pipelines were built, one for each of these experimental modes. The common part, mainly dealing with azimuthal integration, is integrated into a pre-processing pipeline called *integrate multi-frame*.

**Figure 2:**
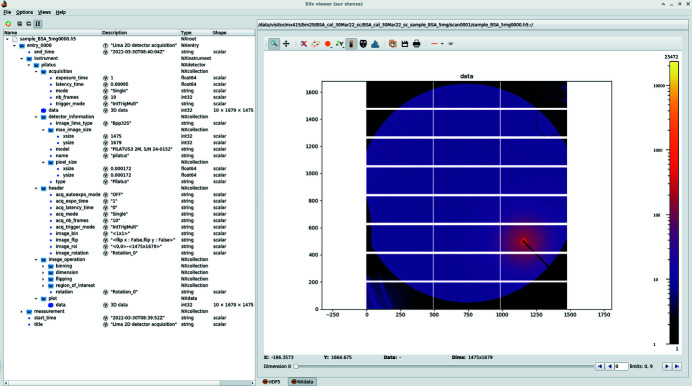
Layout of a raw image HDF5 file produced by the *LIMA* acquisition software and visualized with the *silx* viewer.

Since the Pilatus3 2M detector is controlled by the *LIMA* software (Petitdemange *et al.*, 2018 ▸), raw images are now saved in an HDF5 file format (The HDF Group, 2000–2021 ▸) with ten or 100 frames per file (depending on the acquisition mode, Fig. 2 ▸). HDF5 is not only imposed by the ESRF data policy (Dimper *et al.*, 2019 ▸) but also offers numerous benefits such as compression, faster data-access, symbolic links to data sets stored in other files, *etc.* This data structure with several frames per file prevents us from re-using the former *EDNA* pipelines which were triggered frame by frame.

**Figure 3:**
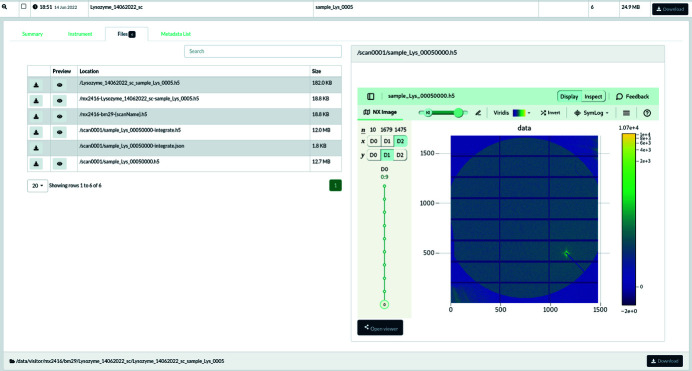
The default visualization offered by the ESRF data portal (https://data.esrf.fr) of a stack of frames acquired at the BioSAXS beamline, using the h5web viewer.

ESRF provides several tools to visualize those HDF5 files; most of them are either based on *silx* (Vincent *et al.*, 2021 ▸), which is a graphical user interface based on *Qt5* (Summerfield, 2007 ▸), or on the web-viewer h5web (Bocciarelli *et al.*, 2022 ▸), visualizing data inside a web browser. The h5web library is already used in the beamline control user interface *BsxCuBE3* (Tully *et al.*, 2022 ▸) and in the ESRF data-portal (ESRF, 2011–2022 ▸) where data are automatically catalogued and made accessible to the experimental team (Fig. 3 ▸) during the embargo period, before making them publicly available, following the ESRF data policy (Dimper *et al.*, 2019 ▸).

Since *LIMA* saves frames containing no metadata beside the camera configuration (Fig. 2 ▸), all sample and experiment description (geometry, mask, *etc.*), beam-stop diode intensities and other processing parameters have to be provided by the experiment sequencer, *BLISS*, as part of the job description when triggering the process. This configuration can be retrieved from the data portal web page (Fig. 3 ▸) as a JSON file (Pezoa *et al.*, 2016 ▸), and is suitable to reprocess the raw data.

The versatility of the HDF5 format allows having one single output file for all results produced by a processing pipeline, making archival easier. Each pipeline registers the result of every individual processing step of the pipeline in the output HDF5 file (as HDF5 groups), together with the configuration associated with each processing. Input data sets are referenced using external links, which avoids data duplication while keeping traceability. Finally, metadata describing the sample, its buffer and the configuration of the beamline are also recorded using the Nexus convention (Könnecke *et al.*, 2015 ▸). Each processing pipeline defines a default plot which tries to summarize the experimental result to the user.

### 3.1. Multi-frame integration pipeline

**Figure 4:**
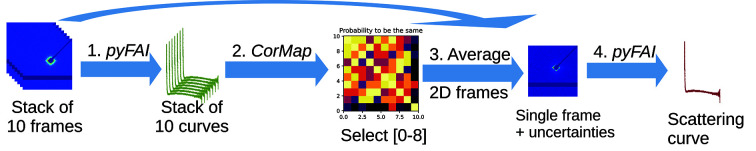
Schematic of the multi-frame integration pipeline: 1, zimuthal integration of individual frames; 2, comparison of 1D curves (the first nine frames are equivalent, the tenth is discarded); 3, equivalent frames are averaged; 4, azimuthal integration of the averaged image.

The multi-frame integration pipeline, Fig. 4 ▸, is triggered with the name of one *LIMA* file (containing several frames) and additional metadata describing the sample and the experiment. This additional information is either collected by the sequencer, *BLISS*, like the beam-stop diode intensity, or read from the user interface, *BSXCube3*, like the sample name and concentration. Samples and experimental conditions for all examples and figures are described in Appendix *A* [App appa].

**Figure 5:**
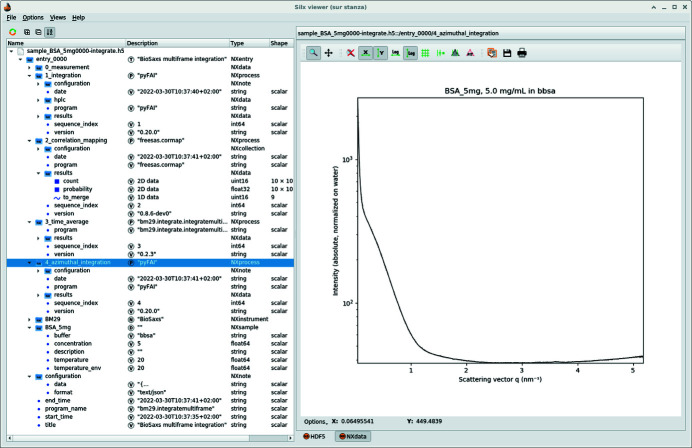
Layout of an HDF5 file obtained from the multi-frame integration pipeline, visualized with the *silx* viewer. The HDF5 tree structure (left-hand side) follows the pipeline described in Fig. 4 ▸. The right-hand-side plot is the averaged scattering curve *I* = *f*(*q*) on a semi-logarithmic scale of the macro­molecule (BSA) before subtraction of the background signal.

Fig. 5 ▸ presents a file produced by this processing pipeline with the default plot consisting of the semi-logarithmic representation of the scattering curve *I*(*q*) viewed with *silx*. This pipeline is built of four subsequent analysis steps, as illustrated in Fig. 4 ▸:

(i) Each recorded image is azimuthally integrated with *pyFAI* (Kieffer *et al.*, 2020 ▸) to produce one scattering curve per frame.

(ii) Scattering curves are compared, searching for radiation damage using the *CorMap* algorithm (Franke *et al.*, 2015 ▸); the probability for each pair of curves to be the same is compared with thresholds to assess their equivalence – those thresholds depend on whether frames were adjacent or not.

(iii) Equivalent images are averaged pixel-wise, weighted by the beam-stop diode intensity; variance is assessed assuming Poisson statistics. Since time-averaging and azimuthal-integration are not commutative (see Appendix *B* [App appb]), the processing restarts from 2D frames and not from individual curves.

(iv) The averaged frame is finally azimuthally integrated and uncertainties propagated accordingly.

The plot of the azimuthally integrated averaged frame (at stage 4) is set as the default display when processing data in sample-changer mode. In HPLC mode, the default plot represents the summed intensity as a function of time, which is a fraction of the complete chromatogram. The HDF5 file additionally includes external links to the raw frames as acquired by the detector (stage 0).

### 3.2. Sample-changer pipeline

In sample-changer mode, solutions containing samples are acquired alternatively with pure buffer solutions. The throughput of the beamline is then limited by the pipetting system of the robot and the delay for cleaning the exposure chamber. The processing is triggered with integrated data from the sample (*i.e.* the name of the file containing the sample data after azimuthal integration) and a list of buffer files corresponding to the different acquisition of buffers, usually the buffer before and the buffer after the sample acquisition.

**Figure 6:**
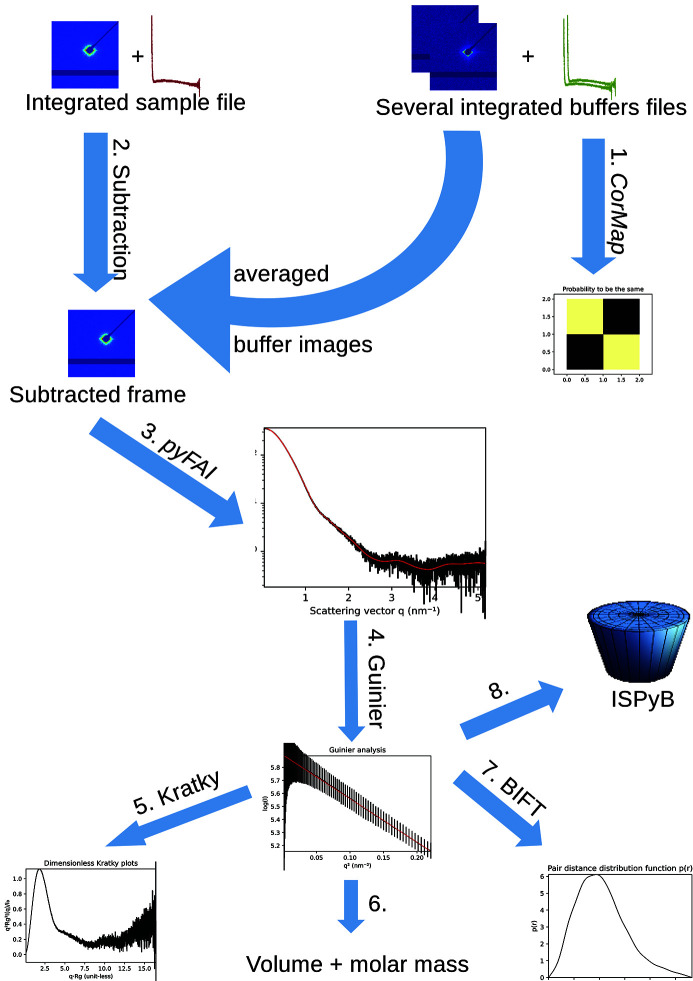
Schematic of the sample-changer pipline: 1, comparison of buffer curves; 2, subtraction of averaged buffer images from sample image; 3, azimuthal integration of the background-subtracted image; 4–7, SAS analysis (contains Guinier fit, Kratky plot and BIFT analysis); 8, registration into ISPyB.

**Figure 7:**
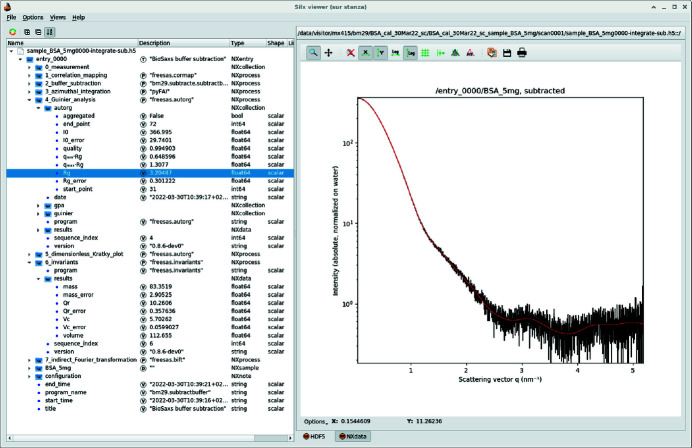
Default visualization of the HDF5 file produced by the sample changer pipeline with the *silx* viewer. The superimposed red curve corresponds to the BIFT modelled data.

The sample-changer pipeline is schematized in Fig. 6 ▸ and produces a new HDF5 file with the subtracted data in it; such a file is visualized in Fig. 7 ▸ and contains the results of this eight-stage pipeline:

(1) Comparison of buffer curves using the *CorMap* algorithm (Franke *et al.*, 2015 ▸).

(2) Buffer frames are averaged together and subtracted from the sample-averaged frame (restarting from 2D raw frames).

(3) Azimuthal integration of the subtracted frame with *pyFAI* (Kieffer *et al.*, 2020 ▸).

(4) Guinier analysis with the associated linear regression of log[*I*(*q*)] versus *q* ^2^ providing *R*_g_ and 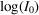 at low *q* as default plot.

(5) Dimensionless Kratky plot: (*q* *R*_g_)^2^ *I*/*I*_0_ versus *q* *R*_g_ to assess the flexibility of the macro­molecule.

(6) Porod (Glatter & Kratky, 1982 ▸) and Rambo–Tainer invariants (Rambo & Tainer, 2013 ▸) calculation to assess the molecular volume and molecular mass of the sample.

(7) Indirect inverse Fourier transform using the BIFT algorithm (Vestergaard & Hansen, 2006 ▸) provides the pair distance distribution function *p* = *f*(*r*).

(8) Transfer of reduced data to ISPyB for BioSAXS (compatibility layer with legacy ISPyB).

As in the *multi-frame* processing pipeline (Section 3.1), there is a link to the source data as stage zero of the processing to ensure a perfect tracking of the experiment. The pair distribution function obtained from BIFT (stage 7) allows calculation of the radius of gyration in real space and should confirm the radius of gyration found from the Guinier fit (stage 4).

Once again, the subtraction is made on 2D frames and not on integrated curves for similar reasons to the one presented in Appendix *B* [App appb].

The final stage of this pipeline is to register those results into the ISPyB for BioSAXS database (De Maria Antolinos *et al.*, 2015 ▸) (https://exi.esrf.fr), and make them instantly available to the user via the data portal (ESRF, 2011–2022 ▸). Once data are registered into the data portal (https://data.esrf.fr), they receive a unique DOI which can be referred to when users are publishing results to the *SASBDB* (Kikhney *et al.*, 2020 ▸). The same data are shared with the *BsxCuBE3* control software via a *memcached* key-value database for instant feedback on the user interface.

### 3.3. SEC-SAXS pipeline

Online purification of the sample prior to measurement enables a reduction in oligomerization. It has become a standard procedure since it was introduced to BM29 in 2012 (Round *et al.*, 2013 ▸) and accounts now for two-thirds of all measurements performed at the beamline.

**Figure 8:**
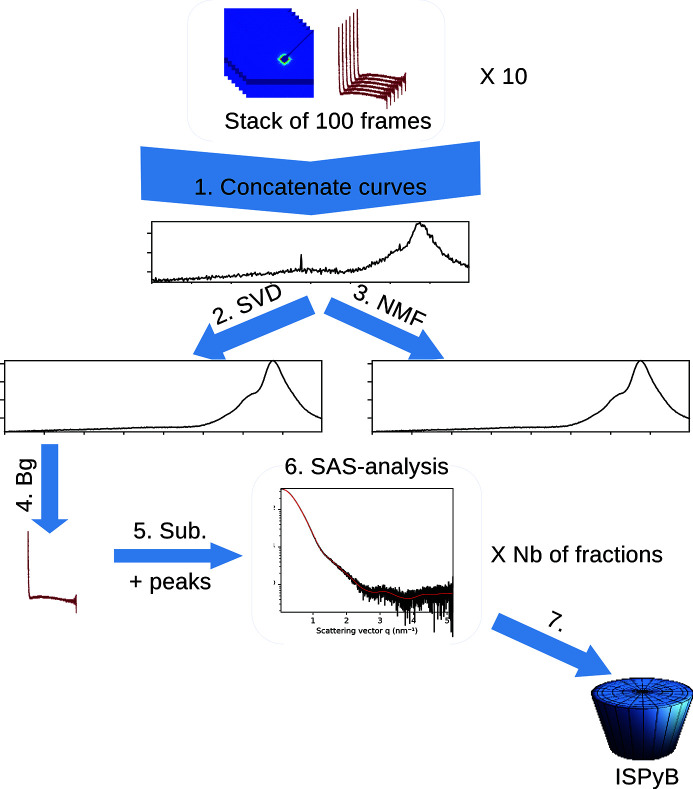
Schematic of the HPLC pipline: 1, concatenate; 2–3, multivariate analysis; 4, background extraction; 5, peak finding; 6, SAS analysis on each fraction; 7, storage to ISPyB.

**Figure 9:**
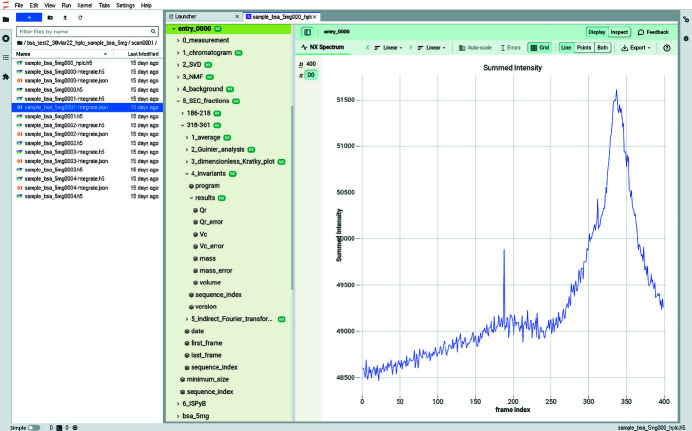
Default visualization of the HDF5 file produced by the HPLC pipeline with the h5web viewer integrated into *JupyterLab*.

In this mode, a typical acquisition consists of 1000 frames saved as ten files containing 100 frames each. The input for this pipeline is a list of HDF5 files with partial chromatograms integrated by the *multi-frame pipeline* presented in Section 3.1. The HPLC pipeline, Fig. 8 ▸, re-builds the complete chromatogram and performs the complete analysis of the different fractions, taking into account the possibility for empty sections (due to missing input files). This pipeline produces files which are presented in Fig. 9 ▸.

This chromatography pipeline has seven processing stages:

(1) Concatenate partial chromatograms (1D curves) provided by the *multi-frame* pipeline to obtain the full chromatogram; empty/missing regions are handled here.

(2) Perform a singlar value decomposition (SVD) on the chromatogram to assess the number of components and extract the scattering from the background (Hopkins *et al.*, 2017 ▸).

(3) Perform a non-negative matrix factorization (NMF) to provide the scattering curve of the different pure components and their associated chromatograms (Bunney *et al.*, 2018 ▸).

(4) Select points belonging to the background by comparing experimental scattering with the first singular vector from the SVD; average selected curves.

(5) Perform peak-picking on subtracted curves to define fractions of the chromatogram.

(6) Analyse each fraction with a similar pipeline to the one presented in Section 3.2: Guinier plot, Kratky plot, BIFT analysis.

(7) Finally, export data and send them to ISPyB for BioSAXS.

Multivariate analysis is performed to extract the signal of the macro­molecule from background scattering, and provides a hint on how many components have been separated in the chromatography. Gavish & Donoho (2014 ▸) provide the number of singular values/vectors which are to be saved after the SVD decomposition The first singular vector contains mainly background scattering, the following ones contain signal from the separated components, and subsequent ones account only for noise. Unfortunately, those singular vectors, beyond the first one, do not look like scattering curves since SVD does not enforce the positivity of those extracted singular vectors during the decomposition (Mak *et al.*, 2014 ▸). Unlike SVD, the NMF factorization is neither fast nor unique, but the extracted components are all positive and look like scattering curves of components. The SVD and NMF algorithms are provided by *NumPy* (Oliphant, 2007 ▸) and *scikit-learn* (Varoquaux *et al.*, 2015 ▸), respectively.

Since none of the multivariate analysis propagates uncertainties, all processing needs to be re-done: a correlation-map is built between the first singular vector of the SVD and all experimental scattering curves. Those curves are ranked from the most likely to be pure buffer to the least likely. Since the major part of the collected fraction are expected to be buffer, the 30% of the curves which are most similar to the first singular vector are considered to be buffer and averaged together at stage 4. Uncertainties are propagated based on deviations calculated during azimuthal integration (and not on 2D frames, see Appendix *B* [App appb]).

The fraction collection (stage 5) is performed on the total scattering chromatogram, smoothed by median filtering. A peak search is performed with the *find_peaks_cwt* function from the *scipy* library (Jones *et al.*, 2001 ▸). It provides a list of regions of high-intensity scattering: the different fractions of the chromatogam. All subtracted curves from the same fraction are averaged and analysed with a similar pipeline as described in Section 3.2: Guinier fit, Kratky plot, various invariant extraction and pair-distribution via BIFT. The results are presented in the same way as in the sample-changer mode, one HDF5 group per fraction.

## 4. Discussions

### 4.1. Statistics

The processing described in Section 3 has been in production since September 2020 and operating for 20 months at the time of writing. Table 2 ▸ summarizes some figures collected.

The run time for *multi-frame* integration presents a clear bi-modal distribution since the same code is used in sample-changer mode (10 frames per acquisition) and in HPLC mode where 100 frames are acquired per file. From the figures in Table 2 ▸, one can estimate that HPLC mode represents 6% of the measurements performed but accounts for 72% of the total measurement time.

### 4.2. Performances

Direct comparison with the former online data-analysis pipeline (Brennich *et al.*, 2016 ▸), based on *EDNA*, is difficult since their structure is very different and the computer equipment has evolved. *EDNA* was processing frame per frame and used to take 2.3 s to integrate a single image from the former Pilatus 1M detector. With this new pipeline, integration occurs at more than 13 frames s^−1^ (Table 2 ▸) with a Pilatus 2M detector.

Performances for the *sample-changer pipeline* (7.3 s) can be directly compared with two of the the former *EDNA* pipelines which were called *autoSub* (2.1 s) and *SAXSAnalysis* (9.7 s).

All computations are now executed on a single computer equipped with a single hexa-core processor and an entry-level graphics card (Intel Xeon E5-2643 v3 + Nvidia Quadro M2000) which have replaced the previous setup (based on Intel Pentium4 + Quadro4000).

### 4.3. Outlook

The foreseeable future should see a better integration of BioSAXS data into the data portal with better web visualization capabilities of the processed HDF5 files.

The buffer averaging and subtraction in HPLC mode is not (strictly) exact since it is based on integrated curves which have been normalized. It should be possible to weight properly those curves to obtain an average which is exactly the same as if one would have averaged or subtracted 2D frames and integrated the result (discussed in Appendix *B* [App appb]). Future algorithmic work will focus on *ab initio* shape reconstruction, based on the DENSS (Grant, 2018 ▸) which is currently too slow to run with real-time constraints at the beamline.

## 5. Conclusion

This document introduces the *FreeSAS* and the *Dahu* software packages which are used, respectively, to analyse BioSAS data and control online data analysis. These two packages are used at the BioSAXS beamline at ESRF, combined with others, to provide complete data-analysis pipelines. The three pipelines described in this contribution have been used in production since 2020, and provide real-time feedback of ongoing experiments to the user. All metadata, all parameters and all references to the source data are recorded together with the processed data into single HDF5 files, which offers not only convenient storage but allows also reproducible science following the FAIR principle. 

## Figures and Tables

**Table 1:** *ATSAS* counterparts to command line programs provided by *FreeSAS*

*FreeSAS*	*ATSAS*
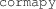	*DATCMP*
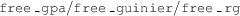	*AUTORG / DATRG*
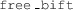	*GNOM / DATGNOM / AUTOGNOM / DATFT*
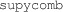	*SUPCOMB / CIFSUP*
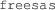	*PRIMUS*
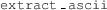	–

**Table 2:** Statistics of the number of job run over 20 months

Processing pipeline	Number of calls	Frames processed	Run-time per job
Integrate multi-frame	42575	1230k	2.1 s (1 s, 10 s)
Subtract and SAS analysis	11214	336k	7.3 s
HPLC analysis	709	893k	2.9 s

## Appendix A Method

All figures were obtained from test samples used at the beamline: bovine serum albumin (BSA) in solution at 5 mg ml^−1^ in a HEPES buffer. The incident X-ray energy was set to 12.5 keV by a multi-layer monochromator with a bandpass of 1% offering a flux of 1.4 × 10^13^ photons s^−1^. Experiments performed in sample-changer mode acquired ten frames of 1 s exposure each. Experiments in HPLC mode used an Agilent Advance Bio SEC 300Å, 4.6 × 300 mm chromatographic column with a flow rate of 0.35 ml min^−1^. Acquisition was performed with 2 s exposure time and lasted 800 s (400 frames).

## Appendix B Rationale for working with 2D frames rather than integrated curves

The *multi-frame* and *subtraction* pipelines perform signal averaging and subtraction on 2D frames rather than azimuthally integrated curves. On the other hand, the HPLC pipeline performs the average and background subtraction on integrated curves.

This section describes the code for performing those two operations and discusses the rationale behind it. It will also try to distinguish which is the most correct. Let  and  be a list of acquired frames and beam-stop diode intensities, respectively. Also assume  is a configured azimuthal integrator (as available from *pyFAI*) and  the number of points in the radial dimension.

### b1. Integrate before averaging

The code can be represent in these four lines of Python code:[Chem scheme1]  Intensities are obtained from an arithmetic average of already weighted curves. Uncertainties are obtained from a quadratic average of uncertainties propagated during integration.

This method does not take into account the fact that some curves did have more weight than others during integration. To exemplify the issue, let us consider the averaging of two buffer data sets: one with 100 frames and the second only with a single frame. Since those buffers are all equivalent, the uncertainties for the first data set will be ten times smaller [(100)^1/2^] than for the second. When merging those data sets with this method, the uncertainties of the first data set are negligible compared with the second, and the propagated uncertainties will be 40% smaller than the second data set, while in reality it should be ten times smaller, [(101)^1/2^]!

As a rule of thumb, the numerical values obtained with this method are correct when all normalization factors are of similar magnitude.

### b2. Sum before integrating

The code can be represented in these three lines of Python code:   and  are equivalent to a single exposure of the detector with much longer integration time, thus a larger normalization factor. This allows the contribution of different frames to be properly weighted during the azimuthal integration [see also equation (5) in Kieffer *et al.* (2020 ▸)].

### b3. Limitations

In sample-changer mode, each pipeline handles only a few dozens images at a time, thus all data can easily be held in the memory of the computer. In HPLC mode, where several hundreds of frames are processed for a single measurement, the same technique could see the computer run out of memory. This is why the HPLC pipeline still averages integrated curves even if it is not strictly exact, but, since all normalization factors are of same magnitude, the result is still correct.

In *pyFAI*, azimuthal integration is performed with ratios of the sum of signal and sum of normalization (Kieffer *et al.*, 2020 ▸). Those sums can be seen as partially processed data and those partial sums can be aggregated to obtain properly weighted average and uncertainties, as described by Schubert & Gertz (2018 ▸). Thus, the memory consumption issue could be alleviated by saving not only the averaged signal but also the sum of signal, the sum of normalization and the partial variances, and base the merging procedure on those figures rather than on the averaged value.
